# Stable Isotope–Assisted Plant Metabolomics: Combination of Global and Tracer-Based Labeling for Enhanced Untargeted Profiling and Compound Annotation

**DOI:** 10.3389/fpls.2019.01366

**Published:** 2019-10-25

**Authors:** Maria Doppler, Christoph Bueschl, Bernhard Kluger, Andrea Koutnik, Marc Lemmens, Hermann Buerstmayr, Justyna Rechthaler, Rudolf Krska, Gerhard Adam, Rainer Schuhmacher

**Affiliations:** ^1^Department of Agrobiotechnology (IFA-Tulln), Institute of Bioanalytics and Agro-Metabolomics, University of Natural Resources and Life Sciences, Vienna (BOKU), Tulln, Austria; ^2^Department of Agrobiotechnology (IFA-Tulln), Institute for Biotechnology in Plant Production, University of Natural Resources and Life Sciences, Vienna (BOKU), Tulln, Austria; ^3^University of Applied Sciences Wr. Neustadt, Degree Programme Biotechnical Processes (FHWN-Tulln), Tulln, Austria; ^4^School of Biological Sciences, Institute for Global Food Security, Queen's University Belfast, Belfast, United Kingdom; ^5^Department of Applied Genetics and Cell Biology (DAGZ), University of Natural Resources and Life Sciences, Vienna (BOKU), Tulln, Austria

**Keywords:** wheat, *Triticum aestivum*, phenylalanine, tryptophan, LC-HRMS

## Abstract

Untargeted approaches and thus biological interpretation of metabolomics results are still hampered by the reliable assignment of the global metabolome as well as classification and (putative) identification of metabolites. In this work we present an liquid chromatography-mass spectrometry (LC-MS)–based stable isotope assisted approach that combines global metabolome and tracer based isotope labeling for improved characterization of (unknown) metabolites and their classification into tracer derived submetabolomes. To this end, wheat plants were cultivated in a customized growth chamber, which was kept at 400 ± 50 ppm ^13^CO_2_ to produce highly enriched uniformly ^13^C-labeled sample material. Additionally, native plants were grown in the greenhouse and treated with either ^13^C_9_-labeled phenylalanine (Phe) or ^13^C_11_-labeled tryptophan (Trp) to study their metabolism and biochemical pathways. After sample preparation, liquid chromatography-high resolution mass spectrometry (LC-HRMS) analysis and automated data evaluation, the results of the global metabolome- and tracer-labeling approaches were combined. A total of 1,729 plant metabolites were detected out of which 122 respective 58 metabolites account for the Phe- and Trp-derived submetabolomes. Besides *m/z* and retention time, also the total number of carbon atoms as well as those of the incorporated tracer moieties were obtained for the detected metabolite ions. With this information at hand characterization of unknown compounds was improved as the additional knowledge from the tracer approaches considerably reduced the number of plausible sum formulas and structures of the detected metabolites. Finally, the number of putative structure formulas was further reduced by isotope-assisted annotation tandem mass spectrometry (MS/MS) derived product ion spectra of the detected metabolites. A major innovation of this paper is the classification of the metabolites into submetabolomes which turned out to be valuable information for effective filtering of database hits based on characteristic structural subparts. This allows the generation of a final list of true plant metabolites, which can be characterized at different levels of specificity.

## Introduction

Untargeted metabolomics studies aim at the detection of as many metabolites as possible. For all living organisms, including plants, the structural diversity as well as wide range of concentrations of the biochemical constituents, prevent one single method from being suited to measure the whole metabolome. The actual size of the metabolome of higher plants remains unknown with estimates ranging from a few thousands (Looser et al., 2005; Aharoni and Galili, 2011) to ca. 50,000 metabolites per individual organism (Pichersky and Gang, 2000; Fiehn, 2002; Hartmann, 2007; Choi Young and Verpoorte, 2014). Wink (1988) estimated that only about 5% to 15% of plant secondary metabolites were analyzed till the time of his publication. Although state-of-the art methods are able to detect hundreds to thousands of plant metabolites simultaneously (Martucci et al., 2014; Doppler et al., 2016), the proportion that can be identified or annotated remains low (Viant et al., 2017).

The identity of metabolites is of utmost interest to be able to convert analytical data into biological knowledge and identification of metabolites is still recognized as the major bottleneck in untargeted metabolomics studies (Tautenhahn et al., 2012; Theodoridis et al., 2012; Wang et al., 2017). In LC-HRMS based approaches, metabolites can only be confidently identified if two or more orthogonal analytical parameters [e.g. retention time (RT), *m/z*, MS/MS fragment spectra] are successfully matched with an authentic reference standard measured within the same sequence at the same instrument (Sumner et al., 2007; Viant et al., 2017; Blaženović et al., 2018). The number of identified metabolites in untargeted metabolomics studies is in general below 50%. Da Silva et al. for example, report 1.8% of the recorded spectra of an untargeted metabolomics experiment to be characterized (da Silva et al., 2015). Usually, a first step toward identification involves a targeted search for known metabolites of the biological organism/the scientific question at hand. The identity of putatively known metabolites can be confirmed by additional measurements if authentic reference compounds are available. However, for the majority of all detected metabolites, their true identity cannot be established with such a targeted search. Based on the accurate *m/z* and the putatively measured ion species of a particular metabolite its sum formula can be postulated and used for subsequent database search. There are heuristics and chemical rules (e.g. nitrogen rule) for such calculations as not all results of simple combinatory methods summing up masses of elements result in meaningful chemical compositions. A valuable help in sum formula calculation is available with the Seven Golden Rules defined by Kind and Fiehn (2007). Recently, sum formula generation has been improved by tools that also take MS/MS fragmentation spectra into account. An example is the SIRIUS software developed by Duhrkop et al. (2019). It generates fragmentation trees and also verifies the isotopolog pattern to assign a score to the generated sum formulas. Unfortunately, even unique sum formulas can represent up to 10,000 different structures (Schrimpe-Rutledge et al., 2016). Various metabolite repositories are (publicly or commercially) available against which the generated sum formulas can be searched. The choice of databases can have substantial influence on the outcome and subsequent interpretation of a study. Small, well defined databases (e.g. a collection of metabolites already described in literature for a certain interaction) can return more meaningful hits compared to searches in gigantic repositories containing several (hundred-) thousand compounds from many different fields that are not related to the scientific and biological question under study (Rochat, 2017). Additional inspection of the suggested metabolites and literature study on their biological role and origin is recommended if numerous database hits appear since the respective metabolites are ambiguously annotated and their structures cannot be taken for granted.

Another form to obtain additional information from each ion are MS/MS fragmentation experiments. MS/MS spectra are characteristic for each metabolite and often allow differentiation between isomers (which would not be possible with sole full scan only). MS/MS experiments are considered as state-of-the art and frequently recorded. Besides targeted MS/MS fragmentation experiments, applying an inclusion list with ions to be fragmented, untargeted automated MS/MS fragmentation experiments allow recording of MS/MS fragment spectra of e.g. the most intense ions. Complementary to these approaches, data independent analysis or SWATH [Sequential Windowed Acquisition of All Theoretical Fragment Ion Mass Spectra (Gillet et al., 2012)] enable fragmentation of broad mass ranges and therefore cover virtually all detected metabolites. With such methods, mixed fragmentation spectra are recorded and spectral cleaning becomes necessary post data acquisition, which can for example be done with the open-source tool MS-DIAL developed by Tsugawa et al. (2015). Thus, obtaining meaningful metabolite-specific MS/MS fragment spectra of sufficient quality on the metabolome-scale in an untargeted manner with the aim to cover as many metabolites as possible is not straight forward. Especially for low abundant metabolites it does not seem to be feasible with the current methods.

The limitations for biological interpretation of not identified metabolites need to be considered if the interpretation is (partially) based on annotations. As these annotations can have different levels of reliability, confidence scales are frequently applied within the metabolomics community (Creek et al., 2014; Schymanski et al., 2014; Schrimpe-Rutledge et al., 2016; Rochat, 2017; Blaženović et al., 2018). Unfortunately, there is currently no community-wide accepted and coherent scheme for metabolite annotation. A first definition of identification levels was presented by the Metabolomics Standards Initiative (MSI) in 2007 (Sumner et al., 2007). Briefly summarized, this work of the MSI defines confidently identified metabolites as level 1 identification, which is based on comparison of at least two orthogonal parameters (e.g. RT, *m/z*, MS/MS fragment spectra) with an authentic reference standard. Level 2 defines putatively annotated compounds (without authentic reference standard measured with the same instrument) based on comparisons of their MS/MS spectra with spectral libraries. Level 3 includes putatively characterized substance classes based on characteristic physicochemical properties of a chemical class, while level 4 is reserved for unknown compounds. Recently, level 0, which comprises the unambiguous 3D structure and includes full stereochemistry identification of an isolated pure compound, was added to the original MSI scheme during the meeting of the 'Compound Identification work group' of the Metabolomics Society in 2017 (Blaženović et al., 2018). Regardless of which metabolite annotation and identification scheme is used, the level of identification together with the used scheme should be stated for each metabolite in a scientific publication as this serves as the basis for further biological conclusions. In the presented manuscript the recently extended annotation and identification scheme according to the MSI, which is widely used in the metabolomics community, is utilized and identification and annotation levels are stated according to Blaženović et al. (2018).

Recently, several novel approaches have been published, which aim at the further, improved and more comprehensive metabolite annotation. Also, more and more MS/MS spectra together with related meta-data are deposited in large repositories for re-use [e.g. MetaboLights (Kale et al., 2016), GNPS (Wang et al., 2016)] and an increasing number of MS/MS reference spectra are being deposited in respective spectral libraries, which can be searched and used as a source for metabolite annotation (e.g. mzCloud (https://www.mzcloud.org), GNPS (Wang et al., 2016), MassBank [(Horai et al., 2010), or more specialized repositories such as the Plant Specialized Metabolome Annotation (PlaSMA) project (http://plasma.riken.jp)]. For example, Heiling et al. used known substances belonging to the class of 17-hydroxygeranyllinalool diterpene glucosides (HGL-DTGs) to compile an MS/MS library and by comparison of fragmentation patterns, more than 100 novel HGL-DTGs were detected in *Solanaceae* (Heiling et al., 2016). With respect to novel data processing tools and workflows, molecular networking, as for example implemented in the GNPS web-application (Wang et al., 2016) or the MS-DIAL software (version 3; Tsugawa et al., 2015; Tsugawa et al., 2019) have attracted attention. These approaches try to find structurally similar compounds based on the assumption that structural similarity also results in similar fragmentation spectra. Clusters of metabolites with similar MS/MS spectra are then enriched with already known compounds. Moreover, identified metabolites can be propagated throughout the MS/MS spectrum network (i.e. to neighboring metabolites or the local metabolite cluster) thereby increasing the confidence in annotation. Moreover, MS2LDA searches for substructures shared between related compounds based on reoccurring fragments. The tool can also use pre-calculated and verified so called Mass2Motifs to search for structurally related compounds in a targeted fashion (van der Hooft et al., 2016; Rogers et al., 2019). Another promising tool, which employs the combined analysis of full scan MS data with MS/MS spectral similarity to find so called metabolite families has been published by Treutler et al. (2016). Only very recently, Fragment Set Enrichment Analysis (FESA) has been published. This novel tool utilizes MS/MS spectra to predict possible metabolite classes of unknown compounds (Tsugawa et al., 2019). Finally, SIRIUS and CSI:FingerID also evaluate MS/MS spectra to improve the prediction of possible sum formulas and to query large compound databases without the need for reference MS/MS spectra (Duhrkop et al., 2019). For more detailed descriptions of these valuable novel approaches the interested reader is referred to the literature.

Stable isotope labeling (SIL) techniques are beneficial for many aspects of the experimental and analytical workflows of untargeted metabolomics approaches. They make use of isotopically labeled (e.g. ^13^C, ^15^N, ^2^H, ^34^S) compounds (exo- or endogenous substances) present in the samples (Hegeman et al., 2007; Giavalisco et al., 2009; Bueschl et al., 2014). This results in characteristic, artificial isotope patterns that can easily be detected with high resolution mass spectrometry. Subsequently, these unique isotope patterns can be used to filter HRMS data for metabolites carrying such a stable isotope label by means of automated software tools.

In this work two approaches, namely global metabolome labeling (GML) and tracer labeling (TL), are utilized and combined to increase the efficiency of untargeted metabolomics approaches and is exemplified by the characterization of the global metabolome as well as by unknown metabolic constituents of wheat.

With GML stable isotopes are introduced to the biological systems via main nutrient sources in order to comprehensively label all endogenously produced metabolites. By using highly enriched, ^13^C-labeled cultures (Hegeman et al., 2007; Giavalisco et al., 2009; Chen et al., 2011; Giavalisco et al., 2011; Bueschl et al., 2014), virtually any metabolite carries the stable isotope label. Beside ^13^C as the most comprehensive stable isotope label, global labeling with ^15^N or ^34^S enriched nutrient solutions (Engelsberger et al., 2006; Gläser et al., 2013) can be used to study the nitrogen or sulfur containing metabolites of plant cell cultures or whole plants. When using LC-HRMS, the characteristic ^12^C^13^C isotope pattern of GML experiments additionally reflects the number of heavy atoms (e.g. carbon atoms in case of ^13^C-labeling) of the respective metabolite (Bueschl et al., 2014), which can efficiently be used to reduce the number of putative sum formulas (Giavalisco et al., 2011).

In the complementary TL approach, isotopically labeled substances of interest are applied to the biological system under investigation. The labeled and also the native form of the substance are then both metabolized. As a result, all downstream metabolites of the tracer compound incorporate the isotopic label or parts of it (Creek et al., 2012; Kluger et al., 2014). Automated data processing allows classification of the detected metabolites as being derived from the previously added tracer. Depending on the biochemical fate of the tracer compound, alterations of the isotopolog pattern can range from slightly broadened isotopolog patterns to complete *m/z* separation of native and labeled analogs.

This work combines GML and TL for the first time with the aim to detect the global wheat ear metabolome as well as the Phe- and the tryptophan (Trp)-submetabolomes. The benefits of the additional information obtained from the labeling experiments will be evaluated and discussed in terms of annotation of unknowns, sum formula generation, interpretation of database search and evaluation of MS/MS fragment spectra. In the related article (Doppler et al., 2019) this approach was used to investigate changes in the Phe-submetabolome of wheat upon treatment with the *Fusarium graminearum* virulence factor and mycotoxin deoxynivalenol.

## Materials and Methods

### Chemicals and Gases

HNO_3_ (65%), NH_4_NO_3_ (≥99%), Na_2_MoO_4_ * 2 H_2_O, KH_2_PO_4_ (≥99.8%), KOH (≥99.5%) as well as LC-gradient methanol (MeOH; LiChrosolv) were purchased from Merck (Darmstadt, Germany). Ca(NO_3_)_2_ * 4 H_2_O, ethylenediaminetetraacetic acid ferric sodium salt (C_10_H_12_N_2_NaFeO_8_), MgSO_4_ * 7 H_2_O, MnCl_2_ * 4 H_2_O, ZnSO_4_ * 7 H_2_O, CuSO_4_ * 5 H_2_O (> 98%) and formic acid (FA, MS grade) were obtained from Sigma-Aldrich (Steinheim, Germany and Vienna, Austria). H_3_BO_3_ (≥99.8%) was obtained from Carl Roth (Karlsruhe, Germany). The uniformly ^13^C-labeled tracer substances ^13^C_9_-phenylalanine (99% isotopic purity) and ^13^C_11_-tryptophan (99% isotopic purity) as well as ^13^CO_2_ (99,14% isotopic purity) were purchased from Eurisotop (St-Aubin, Cedex, France). ELGA water was obtained from an ELGA Purelab Ultra-AN-MK2 system–Veolia Water (Vienna, Austria). ^12^CO_2_ (Gourmet C E290) and synthetic air was purchased from Messer (Gumpoldskirchen, Austria).

Authentic reference standards: chrysoeriol, vitexin, vanillin, trimethyltricetin, homoorientin, and homoeriodictyol were acquired from Extrasynthese (Lyon, France). Sinapaldehyde, cis-aconitic acid, γ-linolenic acid, tryptophan, phenylalanine, p-coumaric acid, kynurenic acid, coniferaldehyde, 5-methoxy-3-indoleacetic acid, chlorogenic acid, caffeic acid, and 2-phenylacetamide were purchased from Sigma-Aldrich (Steinheim, Germany and Vienna, Austria). Tricin glucoside, ferulic acid, azelaic acid, and 5′-deoxy-5′-(methylthio)adenosine were purchased from Fluka (Vienna, Austria). Indole-3-aldehyde was acquired from MP-Biomedicals Inc. (Santa Ana, USA); α-linolenic acid was purchased from Cayman Europe (Tallin, Estonia); 4-hydroxybenzaldehyde was obtained from Merck (Darmstadt, Germany).

### Cultivation of Plants

#### Global Metabolome Labeling

##### Nutrient Solutions

In order to prepare uniformly (U-)^13^C-labeled wheat, plants were grown hydroponically using nutrient solutions adapted from Hoagland and Arnon (Hoagland and Arnon, 1950) and Bugbee (Bugbee, 2004). Nutrient composition was adapted to the growing stages by the successive use of two different nutrient solutions (see **Supplementary Table 1**). The start solution was used until plantlets reached growth stage 11 [BBCH scale, (Zadoks et al., 1974)] followed by the nutrient solution for the vegetative period. Iron was supplied in form of a chelate complex. To prevent ambient CO_2_ being dissolved in the respective nutrient solution, its preparation was started by the addition of nitric acid to achieve a pH <2.5. All other constituents (except KOH) were subsequently added while the solution was continuously homogenized with a magnetic stirrer. After autoclaving and immediately before the nutrient solution was put into the labelbox, its pH was adjusted with KOH to a value between 5.5 and 6.5.

##### Cultivation of Uniformly ^13^C Labeled Plants

^13^C labeling of wheat plants was performed in a customized growth chamber (phytolabelbox) (ECH Elektrochemie Halle GmbH, Halle, Germany) that allows for the control of N_2_/O_2_ (synthetic air) and ^13^CO_2_ content in the atmosphere. Ambient air (i.e. containing ^12^CO_2_) was prevented from entering the phytolabelbox by keeping the system at a slight overpressure of 10 mbar. Prior to positioning of seedlings into the phytolabelbox, seeds of the wheat cultivar Remus were put into blocks of rock wool (Grodan Delta Anzuchtwürfel aus Steinwolle 4 × 4 × 4 cm; Grodan, Roermond, Netherlands) and watered with the start nutrient solution. Germination of seeds was carried out in closed shaded boxes to minimize light exposure. After vernalization at 4°C for 24 h the seedlings were transferred to small containers together with perlite (Thermo-Floor, Natursand; Europerl, St.Pölten, Austria), which was covered with rock wool to prevent alga growth. The containers were transferred into the phytolabelbox and nutrient solution for the vegetative period was added. The atmospheric conditions inside the labelbox were recorded and regulated to CO_2_ 400 ± 50 ppm under light conditions; rel. humidity ≤70%; overpressure of 8 to 10 mbar; in order to save ^13^CO_2_, carbon dioxide was allowed to enrich up to 1,000 ppm in the dark. The parameters for the climate room in which the labelbox had been placed were chosen as follows: days 0–23: 12/12 h day/night cycle with temperatures of 16°C and 12°C respectively. Twenty-four days after placing seedlings into the phytolabelbox, the day/night cycle was adjusted to 14/10 h and temperature was increased to 20°C under illumination and 16°C in the dark. Light was provided by different types of bulbs (Iwasaki NH360FLX, MT400DL BH; Iwasaki, Tokyo, Japan) and photosynthetic photon flux density (PPFD) values between 720 and 1,100 µmol m^−2^ s^−1^ were obtained. Nutrient solution for the vegetative period was added regularly using an external pump. At flowering stage, samples were harvested by cutting whole ears and immediate freezing them in liquid nitrogen. Samples were stored at −80°C until further analysis. For this cultivation 80 L ^13^CO_2_ were consumed to produce 330 g fresh plant material which corresponds to roughly 4 g fresh plant material per liter ^13^CO_2_.

##### Cultivation of Native Plants

Native plants of cultivar Remus were cultivated in parallel to the ^13^C plants in a separate, identical phytolabelbox according to the protocol described above (section Cultivation of Uniformly ^13^C Labeled Plants). However, ^12^CO_2_ was used instead of ^13^CO_2_.

#### Tracer Labeling

Plants of the cultivar Remus were grown in the greenhouse until the flowering stage as described by Warth et al. (2015). At anthesis, 10 adjoining spikelets of a single ear were treated with a total of 200 µl of an aqueous ^13^C_9_-Phe (5 g/L in water) solution. For this, for every spikelet 10 µl of the tracer-solution were applied between the palea and lemma to each of the two primary florets without wounding the plant, corresponding to 1 mg of ^13^C_9_-Phe per ear in total. Three separate ears were treated to obtain replicates. The treated ears were immediately covered with a wetted plastic bag for 24 h to maintain a high humidity. The treated parts of the wheat ear were harvested 72 h after inoculation, immediately frozen in liquid nitrogen and stored at −80°C until further analysis. In parallel the same procedure was carried out for ^13^C_9_-Trp (5 g/L in water) using different wheat ears of the same flowering stage.

### Sample Preparation and LC-HRMS Measurement

**Figure 1:**
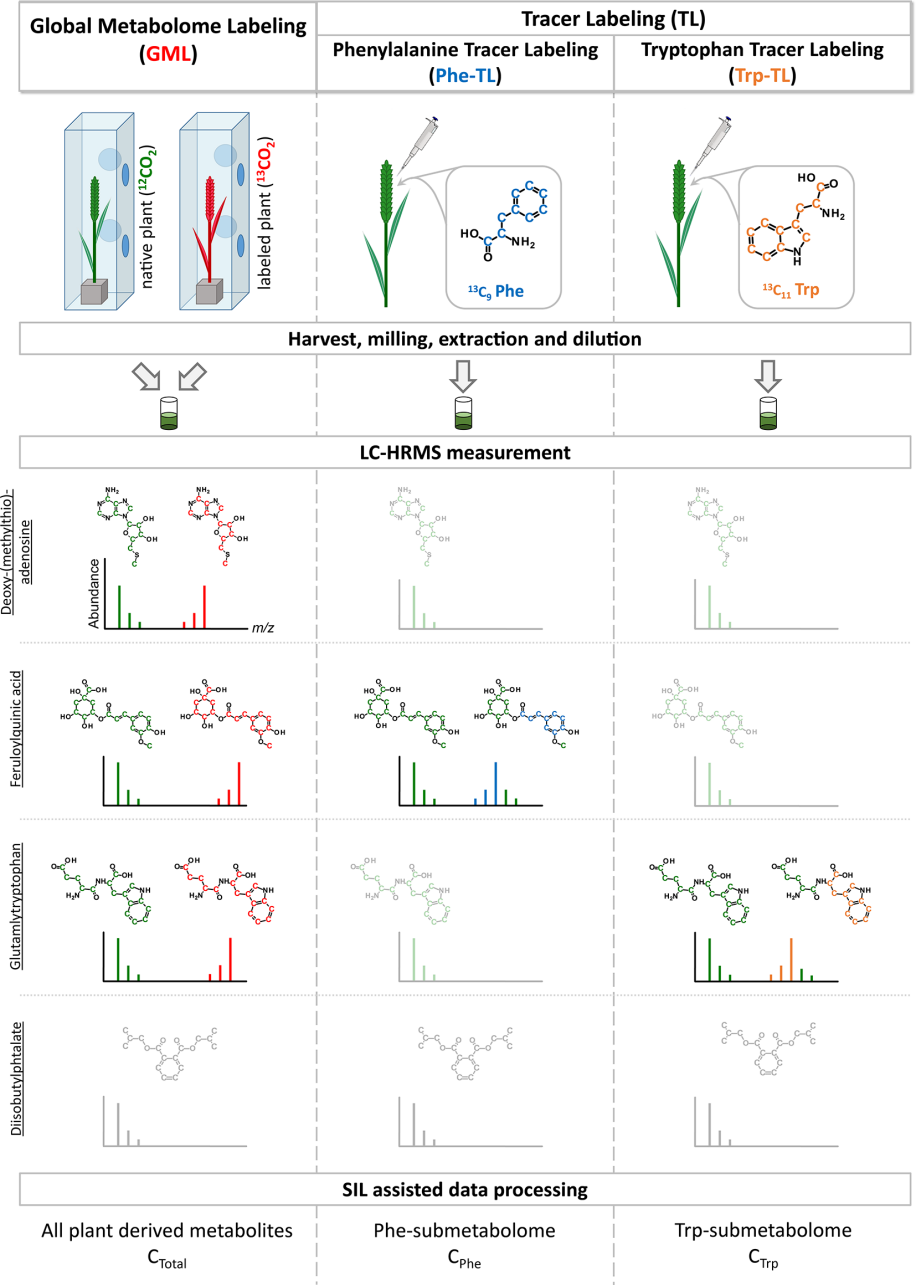
Schematic overview of combining global metabolome- and the two tracer labeling experiments. Example isotope patterns for three truly wheat-derived metabolites are shown. Deoxy-(methylthio) adenosine is a plant metabolite and is therefore detected in the GML experiment. As it is neither a compound of the Phe- or Trp-pathways it is only present as the native isotopic form (shown in green) but not as the tracer-derived isotopically labeled form. On the contrary, feruloylquinic acid is derived from Phe and is subsequently detected as the native and labeled form in the Phe-TL approach as well as in the GML approach. Similar to this, glutamyltryptophan is a metabolite derived from Trp and therefore present as both forms in the Trp-TL and the GML approach. In contrast, diisobutylphtalate is a plasticizer that is frequently detected as a contaminant but is not a plant metabolite and therefore not detected in the SIL-assisted setup.

Frozen wheat ears were ground to a fine powder in pre-cooled 50 mL grinding jars using a ball mill (MM400, Retsch, Haan, Germany). For each sample, 100 ± 2 mg of milled powder were transferred to 2 mL reaction tubes. One milliliter pre-cooled extraction solvent [MeOH:H_2_O 3:1 (v/v) + 0.1% FA; according to (Bueschl et al., 2014)] was added, vortexed for 10 s and subsequently extracted in an ultrasonic bath (Bandelin, Berlin, Germany, Sonorex Digiplus; 640 W, 4°C) for 15 min. Extracts were centrifuged for 10 min at 14,000 rpm at 4°C. For the tracer approaches the centrifuged extracts were diluted with acidified water (+0.1% FA) to obtain a final MeOH:H_2_O ratio of 1:1 [(v/v) + 0.1% FA]. For the GML approach, extracts of the native and globally labeled samples were mixed 1:1 (v/v) and subsequently diluted with acidified water to obtain a MeOH/H_2_O [(v/v), +0.1% FA] ratio of 1:1 (see scheme in **Figure 1**). Five replicates were prepared for the GML- and three replicates for each TL-approach.

The diluted extracts were measured according to the method of Kluger et al. (2014). In brief, 10 µl of extracts were injected and chromatographed via a reversed phase C18 column [XBridge C18 150 × 2.1 mm i.d., 3.5 μm (Waters, Milford, MA, USA)] using a UHPLC system (UltiMate 3000, Dionex). A linear gradient elution with increasing methanol content was utilized. The UHPLC system was coupled to an Exactive Plus Orbitrap system (Thermo Fisher Scientific) via a heated ESI interface operating in fast-polarity switching mode. Mass spectra were recorded in profile mode with a resolving power setting of 70,000 FWHM (at *m/z* 200) and a scan range of *m/z* 100 to 1,000.

LC-MS/MS fragment spectra were recorded with a QExactive HF Orbitrap instrument applying the same chromatographic method as for the full scan measurements described above. MS/MS fragment spectra were acquired in data dependent MS/MS mode following a full scan with resolving power setting of 120,000 FWHM (at *m/z* 200). Spectra were recorded separately for positive and negative ionization mode in two individual measurements. Ten most intense precursor ions were automatically selected by the instrument software based on the antecedent full scan and isolated using an *m/z* window of ±0.75 to prevent M+1 isotopolog ions from being fragmented. Product ion spectra were recorded with normalized stepped collision energy (25, 35, 45 eV) and recorded with a resolving power setting of 30,000 FWHM (at *m/z* 200).

### Data Processing

Raw-full scan LC-HRMS data files were converted to the mzXML format with msConvert from the ProteoWizard toolbox (Kessner at al., 2008) and further processed with the MetExtract II software. The interested reader is referred to Bueschl et al. (2017) for more information about the software’s algorithm.

Briefly summarized, all LC-HRMS data obtained for the GML approach were processed with the AllExtract module of MetExtract II. The software searched for pairs of co-eluting native and U-^13^C-labeled wheat-metabolite ions, with the aim to detect all wheat metabolites. For the Phe-tracer and Trp-tracer treated samples, LC-HRMS chromatograms were processed with the software’s TracExtract module. Here, the software searched for pairs of co-eluting native and partly ^13^C-labeled wheat-metabolite ions, which contained one or more (intact) ^13^C-labeled tracer-derived moieties or derivatives thereof using tracer specific parameter settings. For each of the three sample types, several solvent- as well as native wheat derived LC-HRMS data files were processed as native-blanks to estimate the number of false-positive results. A detailed list of all data processing parameter settings used for each experiment is provided in **Supplementary Table 2**.

### Combination of Global and Tracer-Based Labeling

Data processing by MetExtract II resulted in three separate lists of respective wheat metabolites (i.e. derived from GML, Phe-, and Trp-labeling respectively), which were subsequently merged into one data table by a custom software script implemented in the Python programming language (https://www.python.org). To this end, the *m/z* value of the monoisotopic, native isotopologs as well as their RT, ionization mode and charge number were compared across the three experiments. A maximum *m/z* deviation of ±5 ppm and a maximum RT shift of ±0.15 min was allowed for the respective features to be merged into a single metabolite/metabolic feature of the reference list. The determined number of ^13^C-atoms of each experiment was not considered during the merging step as this property is dependent on the labeling approach (i.e. uniformly or partly ^13^C-labeled ions respectively).

The output of this strategy is a comprehensive list of detected wheat-derived ions. Moreover, ions belonging to the same metabolite such as adducts, in-source fragments, or polymers are also automatically convoluted into metabolites. For each detected metabolite ion, its total number of carbon atoms, its number of carbon atoms derived from the Phe- or Trp-tracers, *m/z*, RT, charge number, ionization mode, and (if available) the assigned ion species (e.g. protonated or deprotonated ions) or common in-source fragment (e.g. -H_2_O), as well as an identifier to which metabolite (i.e. feature group) a certain ion belongs, are reported.

### Target Search, Identification, Sum Formula Generation, and Annotation of Metabolites

**Figure 2:**
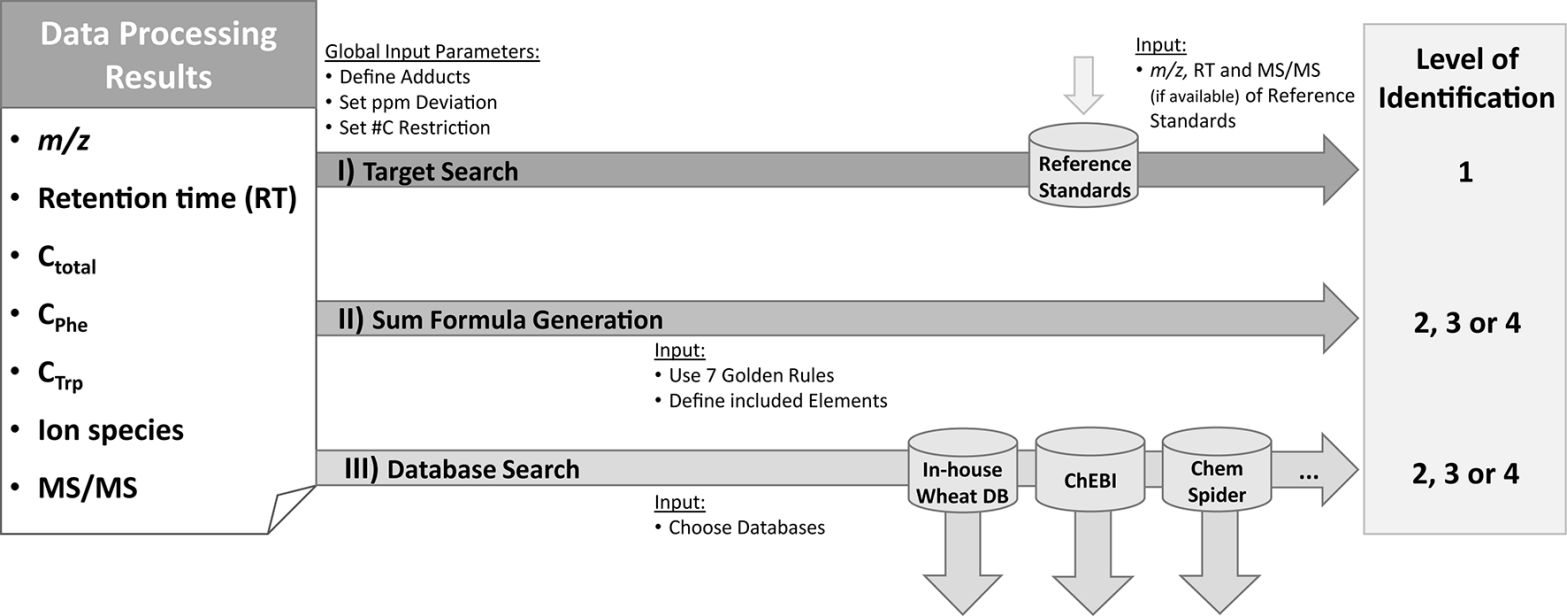
Identification and annotation workflow. Based on the information from the raw data [*m/z* and retention time (RT), availability of MS/MS spectra] as well as from MetExtract II evaluation [#C in total (C_total_); from Phe-TL (C_Phe_) or Trp-TL (C_Trp_); ion species] three steps are performed for each detected metabolite. For target search, accurate *m/z* and RT of the metabolite are compared with authentic reference standards measured in the same sequence and the same analytical method. This step can result in level 1 identification. In the second step, sum formulas are generated using the Seven Golden Rules algorithm (Kind and Fiehn, 2007). The third step consists of database search based on the *m/z* and the known number of carbon atoms for each metabolite. Identification levels are based on the Metabolomics Standards Initiative (Blaženović et al., 2018). ChEBI database (Hastings et al., 2013); ChemSpider database (Pence and Williams, 2010)

After the results of all three approaches had been merged into a single data table, metabolites were identified based on comparison with authentic reference standards. Putative sum formulas were generated according to the Seven Golden Rules (Kind and Fiehn, 2007) and database searches were performed. A scheme giving an overview of these steps is depicted in **Figure 2**.

Metabolite identification was achieved by comparing RT, *m/z*, C_n_ from the GML experiment, and, if recorded, MS/MS fragment spectra of the detected compounds with authentic reference standards. This comparison allows level 1 identification, which is defined as successful comparison of at least two independent orthogonal parameters (e.g. MS, MS/MS, RT) of the metabolite of interest with an authentic reference standard measured under the same conditions (i.e. the same instrument and the same method) (Blaženović et al., 2018). As a starting point for sum formula generation the Seven Golden Rules using C, H, N, O, S, and P were applied (Kind and Fiehn, 2007). Only those putative sum formulas that had the same number of carbon atoms as determined in the GML approach were accepted. Subsequently, the detected metabolites were searched for in a wheat-specific in-house metabolite database collected from various sources (literature reports, PlantCyc (Schläpfer et al., 2017), Combined Chemical Dictionary (http://ccd.chemnetbase.com/faces/chemical/ChemicalSearch.xhtml; last accessed November 2018) and Phenol Explorer (Rothwell et al., 2013; currently consisting of >1100 entries) and the public repositories ChEBI (Hastings et al., 2013) and ChemSpider (Pence and Williams, 2010). For this, the *m/z* values of the detected monoisotopic ions together with their assigned total number of carbon atoms were searched against the respective database entries. For both the sum formula generation and the database queries a maximum *m/z* deviation of ±5 ppm was allowed. The most commonly formed adducts ([M+H]^+^, [M+NH_4_]^+^, [M+Na]^+^, [M+K]^+^, [M+CH_3_OH+H]^+^, [M-H]^−^, [M+Na-2H]^−^, [M+Cl]^−^, [M+Br]^−^) were considered for calculating the putative mass of the metabolite from its respective ion whenever the mass of the non-charged intact metabolites could not be derived from the MS spectra (e.g. when only one ion species of the metabolite was detected). Any metabolite ion that was only detected in the Phe- or the Trp-tracer experiment and for which the total number of carbon atoms was thus unknown (no match in the GML approach), the determined number of tracer-derived carbon atoms was used as the minimum number of carbon atoms for generating sum formulas and querying the compounds against the databases. Sum formula generation and data base query resulted in the identification levels 2, 3, or 4 (Blaženović et al., 2018).

### MS/MS Spectrum Evaluation

MS/MS fragment spectra were manually inspected using the XCalibur software (Thermo Scientific, version 4.0.27.19). If both the native and the ^13^C-labeled forms of an ion were fragmented (e.g. [M+H]^+^ native form, GML form and/or tracer-labeled form) automated MS/MS fragment spectra evaluation by the FragExtract module of MetExtract II was employed. Parameter settings are listed in **Supplementary Table 2**. Additionally, a search in the mzCloud database (https://www.mzcloud.org) was conducted (similarity cutoff 85; HighChem HighRes matching algorithm).

## Results

### Overview of Generated Results

**Figure 3:**
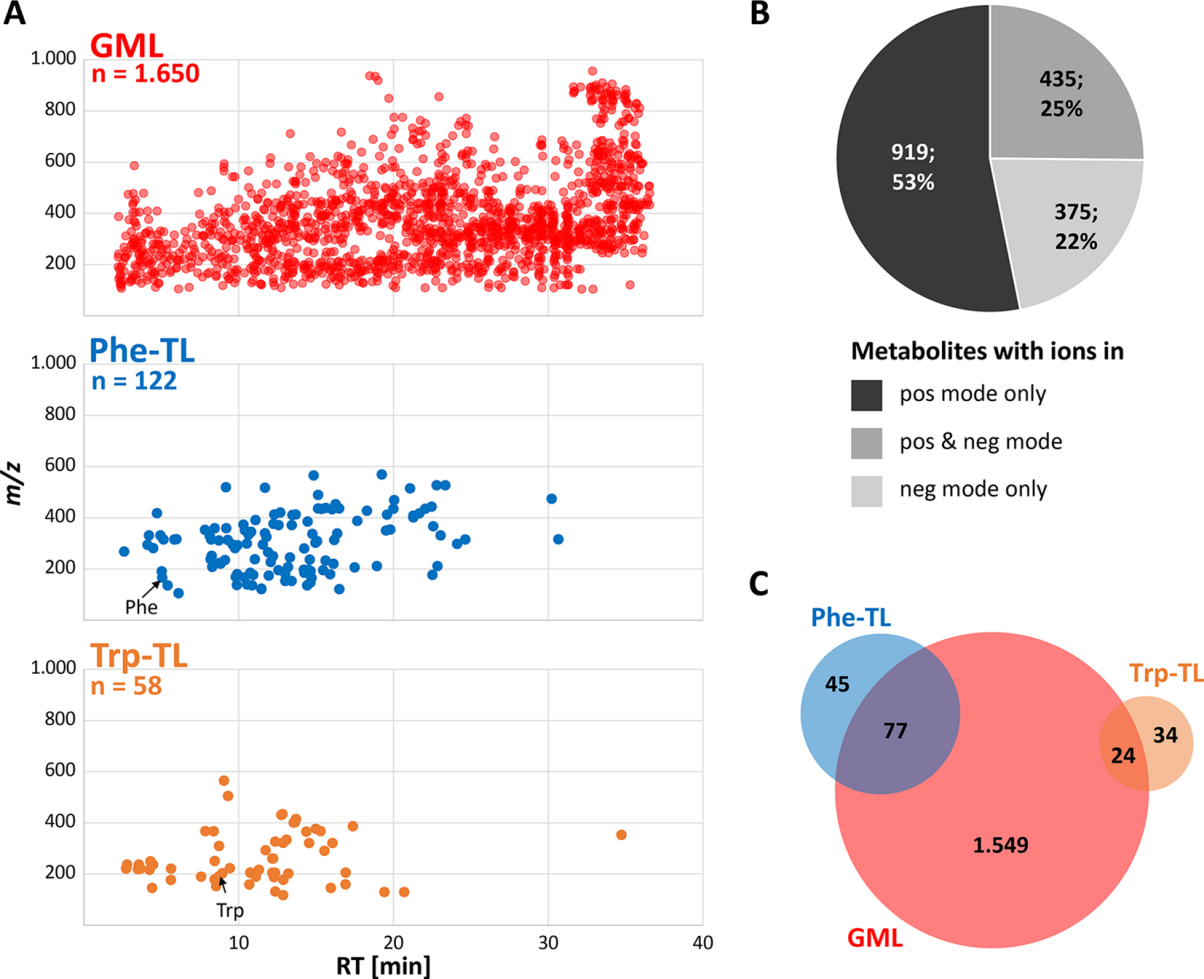
Overview of detected wheat metabolites. **(A)** *m/z* vs. retention time feature maps of the detected metabolites. The most abundant monoisotopic ^12^C feature per metabolite is depicted for the GML (red), Phe-TL (blue) and Trp-TL (orange) experiments; **(B)** number and percentage of metabolites detected as ions in either the positive or negative or both ionization modes; **(C)** overlap of the global wheat- Phe-, and Trp-derived metabolites after combining data of the separate approaches.

Global metabolome labeling of wheat plants in the phytolabelbox resulted in plant material with uniform ^13^C isotopic enrichment of approximately 98.6%, which was well suited for the GML approach and automated data processing with AllExtract. Moreover, the two applied tracer compounds Phe and Trp were successfully taken up and metabolized by the treated wheat ears resulting in numerous Phe- and Trp-derived compounds in the respective sample types. LC-HRMS measurements in fast polarity switching mode and subsequent evaluation with MetExtract II resulted in a total of 4,669 feature pairs (i.e. metabolite ions) corresponding to 1,729 plant metabolites. Thereof, 1,650 metabolites (4,570 feature pairs) represent the metabolome obtained for the globally ^13^C-labeled wheat ear samples. The tracer approach applying Phe resulted in a submetabolome of 122 metabolites (795 feature pairs) and the Trp submetabolome consisted of 58 compounds (254 feature pairs). An overview of the detected metabolites is shown in **Figure 3**.

The detected wheat metabolites were almost evenly spread in both the RT and *m/*z dimension, which reflects the large variation in polarity and molecular mass respectively (**Figure 3A**). With a few exceptions the Phe- and in particular the Trp-derived metabolites constitute rather polar to mid-polar compounds of ≤*m/z* 600 as can be seen from their elution at earlier RTs. Roughly, half of the metabolites (53%) were only detected in positive ionization mode (**Figure 3B**) whereas 22% of the metabolites were solely found in negative ionization mode. A quarter of the metabolites were detected in both ionization modes. For the two submetabolomes a lower percentage of metabolites was detected with the negative ionization mode only (16% Phe-TL and 12% Trp-TL) whereas more metabolites of the submetabolomes were detected with both ionization modes (52% for Phe-TL and 33% for Trp-TL).

The complete list of detected metabolites is provided in the **Supplementary Table 3**.

### Characterization of Detected Metabolites and Exemplification of Metabolite Annotation

**Figure 4:**
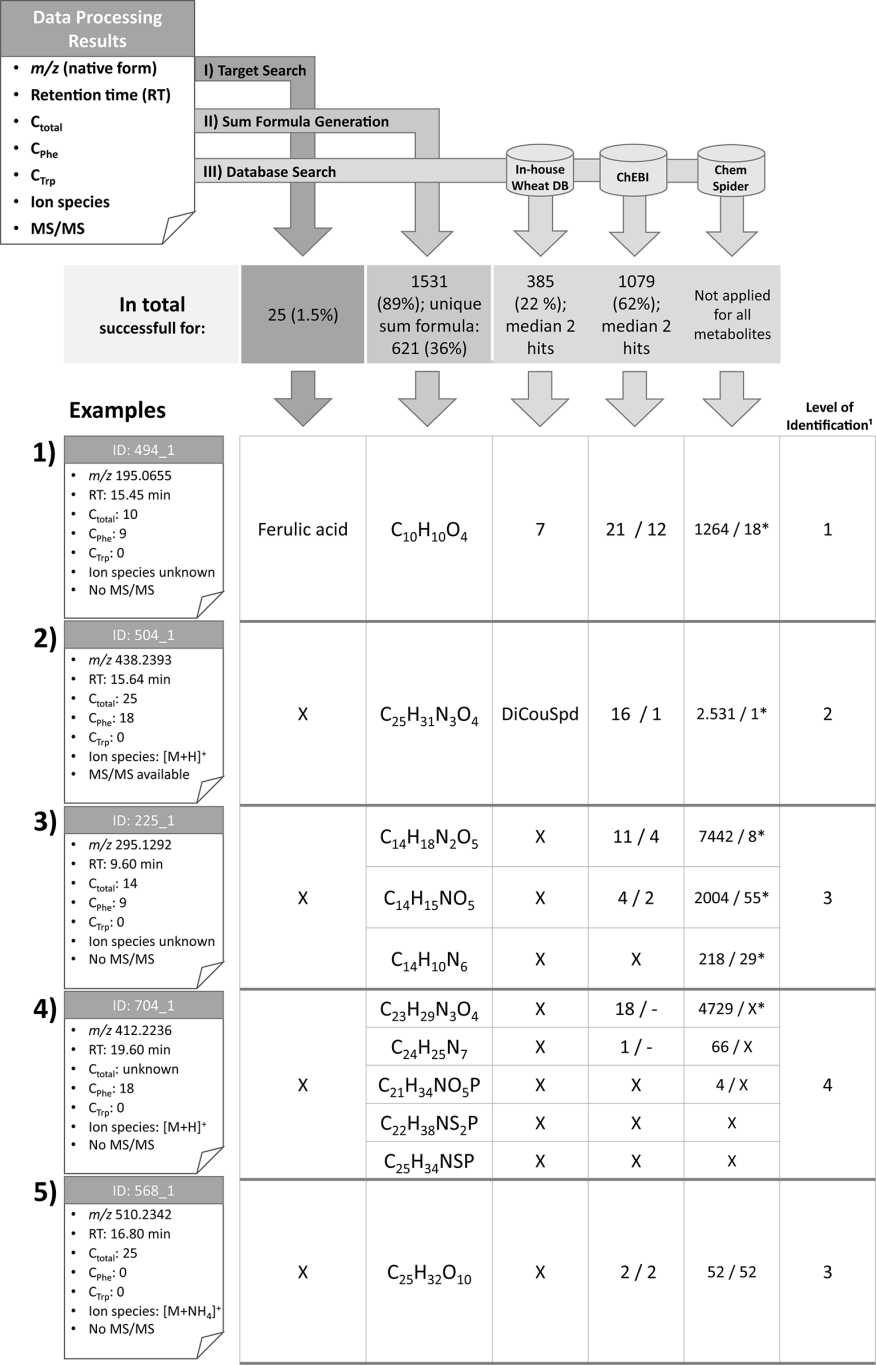
Overview of the identification and annotation scheme for the detected metabolites and illustration for five selected wheat compounds (1–5). Based on MetExtract II evaluation three steps were performed for each metabolite: I) target search, II) sum formula generation, and III) database search. ¹: Identification levels are based on the Metabolomics Standards Initiative (Blaženović et al., 2018); DB: database; X: no results; *: number of plausible results within the first 100 database hits; DiCouSpd: dicoumaroylspermidin.

For further characterization of the detected metabolites, their RT, *m/z* as well as their total number of carbon atoms, assigned ion species, and MS/MS information as well as authentic reference standards were used for metabolite identification and annotation according to **Figure 2**. Examples of this process are exemplified in **Figure 4**.

For 25 metabolites the comparison with authentic reference standards was successful and facilitated level 1 identification (Blaženović et al., 2018). The following metabolites could be identified (for each metabolite the IDs are provided. Since several ions have been identified, the IDs are shortened since each metabolite may have multiple ions that all have been identified by the comparison): 2-phenylacetamide (ID 266), 4-hydroxybenzaldehyde (ID 294), 5′-deoxy-5′-(methylthio)adenosine (ID 201), 5-methoxy-3-indoleacetic acid (ID 578), α-linolenic acid (ID 1576), azelaic acid (ID 698), caffeic acid (ID 325), chlorogenic acid (ID 267), chrysoeriol (ID 926), cis-aconitic acid (ID 18), coniferaldehyde (ID 515), ferulic acid (ID 494), γ-linolenic acid (ID 1388), homoeriodictyol (ID 798), homoorientin (ID 486), indole-3-aldehyde (ID 516), kynurenic acid (ID 279), p-coumaric acid (ID 463), Phe (ID 113), sinapaldehyde (ID 518), tricin glucoside (ID 643), trimethyltricetin (ID 1043), Trp (ID 198), vanillin (ID 373), and vitexin (ID 521). Based on the Seven Golden Rules (Kind and Fiehn, 2007) it was possible to generate sum formulas for 1,531 metabolites (89% of all metabolites), with 621 (36%) metabolites having been annotated with a single sum formula. The knowledge of the total number of carbon atoms enabled to remove approximately 4/5 of putative sum formulas generated by the Seven Golden Rules. This effect is even more obvious for metabolites with higher masses. Knowledge of the metabolites’ ions species also lowers the number of potential sum formulas (see **Supplementary Figure 1**).

For 385 metabolites (22%) the search in the in-house wheat metabolite database resulted in one or more hits with a median of two database hits per detected metabolite. For 1,079 measured metabolites (62%) search against ChEBI was successful. These searches returned a median value of two database hits per metabolite. As the ChEBI and ChemSpider databases consist of numerous non-wheat chemicals, search against those databases resulted in many hits, the majority of which can be discarded. Manual inspection of the database hits, especially for the metabolites carrying tracer moieties, was necessary. Selected examples for annotation/identification are shown in **Figure 4**. Only the first 100 hits displayed on the website were checked manually for chemical plausibility (only selecting structures carrying the tracer moieties (e.g. the C_6_–C_3_ unit), if nine carbon atoms from the Phe tracer were detected). The ChEBI database was searched for all detected metabolites. Moreover, the ChemSpider database was used to annotate selected representative metabolites only.

In the following, the complementary and added value of combining GML and TL for metabolite annotation/classification are exemplified representing different levels of identification (Blaženović et al., 2018). Nevertheless, all detected and annotated metabolites of this manuscript are presented in **Supplementary Table 3**. In view of the related, biological study (Doppler et al., 2019), which investigated the Phe-related defense response of wheat against the fungal pathogen *F. graminearum*, we have selected five respective and representative metabolites to exemplify our isotope-assisted annotation approach.

Metabolite 494, which is represented by the ion with the ID 494_1 (example 1) is the known wheat metabolite ferulic acid for which comparison of RTs and manual verification of the raw data resulted in identification level 1 (Blaženović et al., 2018). As Phe is a biochemical precursor for ferulic acid, the compound was detected in both the GML and Phe-TL experiments. It consists of nine carbon atoms from the tracer Phe, which is in agreement with the pathway from Phe to ferulic acid (via cinnamic, p-coumaric, and caffeic acid). As this metabolite was identified in the first step of the identification scheme, further database search and sum formula generation were omitted. However, for the sake of completeness the corresponding results are also illustrated in **Figure 4**.

For metabolite 504 it was possible to predict its exact mass via its ions [M+H]^+^ (ID 504_1) and [M-H]^−^ (ID 504_11). Moreover, it was assigned a total of 25 carbon atoms, 18 of which originated from the Phe tracer, which indicates the incorporation of two full Phe moieties. Comparison against authentic reference standards available for this measurement did not give any results. Sum formula generation resulted in C_25_H_31_N_3_O_4_. Search against the in-house wheat database resulted in one potential hit—the dihydroxycinnamic acid amide dicoumaroylspermidine (DiCouSpd). The ChEBI database gave 16 and ChemSpider 2,531 results respectively. As this metabolite contained two C_6_–C_3_ moieties the structures of the database hits were screened accordingly. Within the 16 hits from ChEBI only one structure contained two C_6_–C_3_ units, namely CHEBI:85530; N^1^,N^8^-bis(coumaroyl)spermidine. Also, manual inspection of the first 100 hits from ChemSpider search resulted in a single structure with two C_6_–C_3_ moieties (ChemSpider ID 27471687). All three selected database hits represent the same metabolite. As for this metabolite no reference standard is available, the metabolite's annotation level is 2 (Blaženović et al., 2018).

Metabolite 225, represented by feature 225_1 consists of 14 carbon atoms with 9 deriving from Phe. The true identity of the metabolite could not be verified using the authentic reference standards. The ion species of the compound remains unknown as no common mass increment between the detected ions was found. Three putative sum formulas containing 14 carbon atoms were generated but no database annotations were possible with the in-house wheat database. For two of three sum formulas search against ChEBI was successful (11 and 4 hits). For all three sum formulas numerous hits were obtained from ChemSpider search. Limiting the hits to structures fulfilling the Phe-tracer criterion 4 respectively two hits for ChEBI and 8/55/29 out of the first 100 ChemSpider results remained. Thus, multiple sum formula and structure options remain for this metabolite, which can be assigned to annotation level 3 (Blaženović et al., 2018).

Metabolite 704 contains 18 Phe-TL derived C atoms, while the total number of carbon atoms remains unknown since the metabolite was only detected in the Phe-submetabolome experiment. Use of the authentic reference standards did not yield the true identity of the compound. The ion species was annotated to be [M+H]^+^ (ID 704_1) since the corresponding [M-H]^−^ ion (ID 704_2) has also been detected in the negative ionization mode. Based on this knowledge, five different sum formulas with different numbers of carbon atoms (21, 22, 23, 24, and 25) were generated. While annotation against the in-house wheat metabolite database did not return any results, 19 respective hits were generated for ChEBI whereby none of the proposed structures contained two C_6_–C_3_ units. Also for ChemSpider search none of the first 100 suggested structures fulfilled the Phe-tracer criterion. According to the identification scheme suggested by the MSI (Blaženović et al., 2018) this metabolite remains unknown (i.e. level 4 annotated). Without the help of the Phe TL experiment, this metabolite would have been incorrectly annotated with level 2 (plausible database hit).

The fifth example describes a plant metabolite that was not annotated as a Phe- or Trp-derived wheat compound and has 25 carbon atoms in total. From the detected ions it was possible to calculate the metabolite's accurate mass since two ions have been assigned with adducts [[M+NH_4_]^+^ (ID 568_1) and [M-H]^−^ (ID 568_6)] and a unique sum formula. ChEBI database search resulted in two hits and a search in ChemSpider yielded 52 hits. As there is no information concerning tracer-derived subunits of the metabolite all database hits are plausible. The compound is therefore identified with level 3 (Blaženović et al., 2018).

**Supplementary Table 3** lists all detected metabolites and the information from raw data and MetExtract II evaluation as well as the number of database hits and if <15 hits also their names and IDs.

### MS/MS Fragment Spectra Interpretation

**Figure 5:**
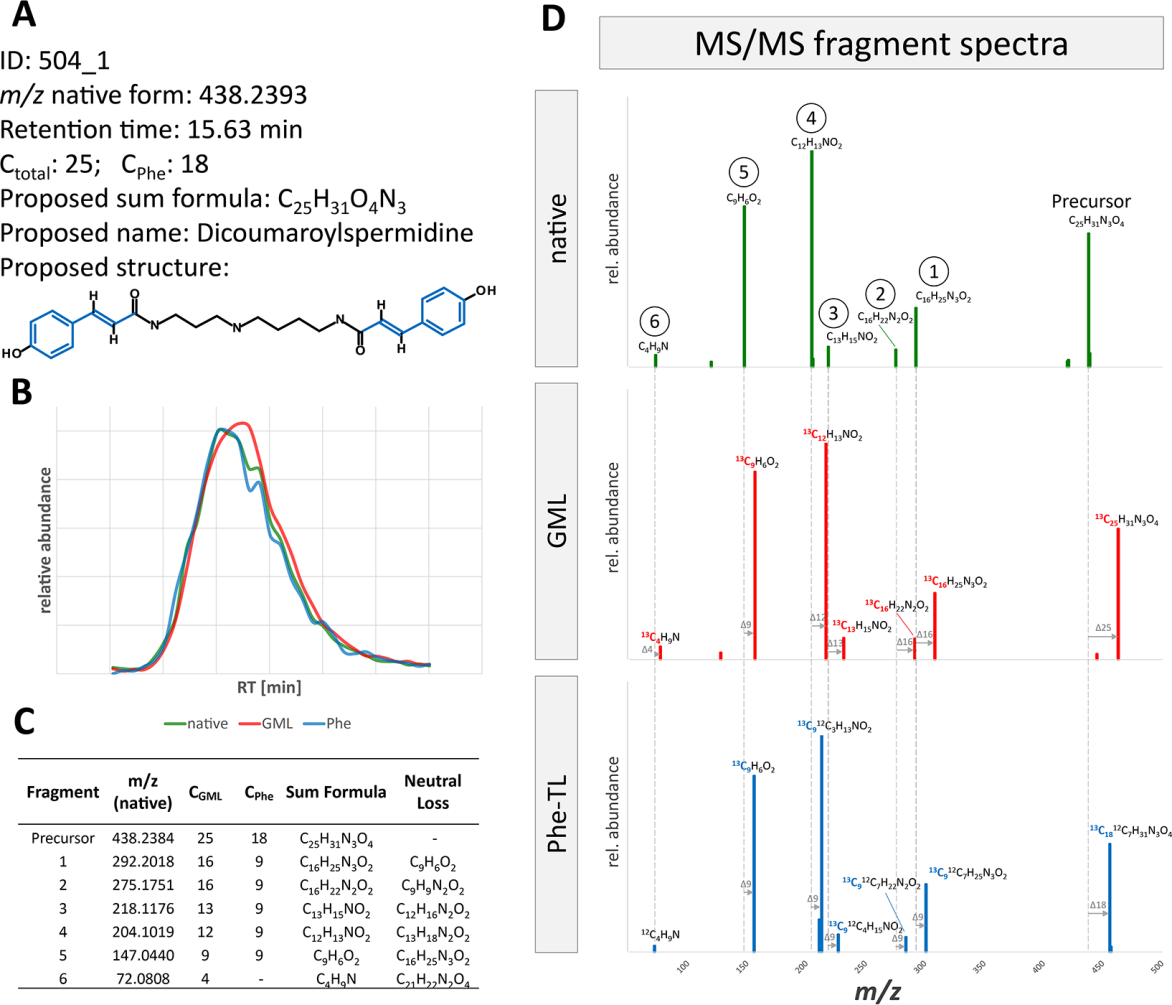
MS/MS fragment spectra interpretation for metabolite ion 504_1. **(A)** Information gained from raw data, MetExtract II evaluation, sum formula generation and database search. **(B)** Extracted ion chromatograms of the precursor ion of the native (green), uniformly ^13^C-labeled (red, from GML) and partly ^13^C-labeled (blue, from Phe-TL) forms from the different approaches. **(C)** List of fragments from FragExtract data evaluation (i.e. automatically matched fragment ions between the native and ^13^C-labeled metabolite forms); **(D)** MS/MS fragment spectra of the differently labeled metabolite forms.

Annotated metabolites can be further characterized by their MS/MS fragment spectra. For this, MS/MS product ion spectra of the differently labeled forms were recorded. MS/MS fragment spectra were obtained using a data dependent MS/MS method with a QExactive HF instrument. In total, MS/MS fragment spectra could be recorded for about 20% of all MetExtract II derived metabolite ions (20% in GML, 28% in Phe-TL, 18% in Trp-TL). All recorded MS/MS fragmentation spectra are provided in **Supplementary Datasheet 1**. **Figure 5** exemplary shows the MS/MS spectra of feature 504_1, which was annotated as [M+H]^+^ of DiCouSpd (**Figure 4**) together with those of its labeled analogs from GML (*m/z* 463.3231) and TL (*m/z* 456.2996). MS/MS library search in mzCloud did not result in any hits for this compound.

The proposed structure obtained from the database annotations of DiCouSpd served as the target for the subsequent automated MS/MS fragment evaluation (**Figure 5**). Considering native and ^13^C-labeled precursor as well as fragment ions, the FragExtract module of the MetExtract II software package was used to automatically match and annotate six fragments. For each ion the total number of carbon atoms as well as the moiety of the applied Phe tracer can be evaluated by the mass increment between the native fragment and the differently labeled analogs. The observed fragments are in good agreement with the proposed structure. For example, fragment 5 represents a characteristic fragment of coumaric acid, a Phe-derived wheat constituent, which is incorporated two times in the metabolite. It has nine carbon atoms in total, all of them originating from Phe which can be seen in the MS/MS spectra of the partly ^13^C-labeled compound in the GML and Phe-TL experiment. This fragment was also present in the MS/MS spectrum of the authentic reference standard of coumaric acid (data not shown).

The spectral information obtained from the GML- and TL-experiments and the respective annotations can be combined with many other annotation approaches that utilize MS/MS (or MS^n^) fragmentation patterns such as FESA (Tsugawa et al., 2019), molecular networking (GNPS; Wang et al., 2016), or substructure Mass2Motif search (van der Hooft et al., 2016). The interested reader is asked to refer to these tools for their complementary use in metabolite annotation.

## Discussion

### Global Metabolome/Overlap With Submetabolomes

As has been described earlier, the presented global labeling approach allows a holistic description of the metabolic inventory of the studied wheat ears (Kluger et al., 2014; Doppler et al., 2016). The unambiguous assignment of a total of 1,729 metabolites was only possible with the high specificity introduced by the labeling specific isotope patterns. Combining global labeling and TL proved to be particularly valuable for those metabolites, which are located downstream of the investigated tracer compounds Phe and Trp. The tested endogenous tracers Phe and Trp resulted in submetabolomes consisting of 122 (Phe) and 58 (Trp) metabolites, of which 77 (Phe) respective 24 (Trp) were also detected in the GML experiment.

Complementary to the shared constituents, 45 (Phe), and 34 (Trp) metabolites were only detected in the tracer but not in the GML experiment. Manual inspection revealed that the majority of these metabolites are also present in the GML samples but at lower concentrations and thus were potentially below the abundance threshold set for automated data evaluation. Likely reasons for this are that different growing conditions between the phytolabelbox (GML) and the glasshouse (TL), slight differences in the growing stages or daytime of harvest had an effect on the metabolic composition of the plants deriving from the two different cultivation regimes (Pyl et al., 2012; De Souza et al., 2015). Moreover, application of the tracer substance can result in the activation of metabolic pathways situated downstream. For example, kynurenic acid, which is known to be a degradation product of Trp was only detected in the TL experiment but not in the GML samples.

On the other hand, it is quite likely that some metabolites of the GML experiments, which do not have any pendants in the respective Phe- or Trp-TL experiments, are in fact Phe- or Trp-derived compounds. For example, metabolites like homoeriodictyol or chrysoeriol, which were only found in the globally labeled samples, may have been formed during early development of the plant but not after application of the labeled tracer and can therefore not be detected by the used tracer approach. In addition, intermediates of high flux carrying pathways may be present at very low levels and therefore be missed by either of the two approaches. Examples of this would be sinap- or coniferaldehyde, which are known to be reactive Phe-derived intermediates during lignan and lignin biosynthesis.

In the presented study, the Phe- and Trp-derived submetabolomes do not overlap. However, this does not mean that wheat does not produce such metabolites consisting of Phe and Trp derived moieties but rather that they have not been produced by the plants from the ^13^C tracers in sufficient amount in the observation period of 72 h. Examples of this would be the class of hydroxycinnamic acid amides (HCAAs) with feruloylserotonin as a metabolite that is known to be involved in the defense against pathogens (Gunnaiah et al., 2012).

### Enhanced Evaluation of Detected Metabolites

#### Generation of Sum Formulas

The dataset presented here, allowed sum formula prediction for 89% of the detected metabolites. While also conventional LC-HRMS based approaches make use of the isotopic envelope of compounds for sum formula generation (e.g. the ratio of the monoisotopic and the first isotopolog mass peak provides information about the approximate number of carbon atoms in the metabolite), the labeling-derived knowledge of the metabolites' total number of carbon atoms per formula unit offers additional benefit for sum formula generation (**Supplementary Figure 1**). Furthermore, GML with stable isotopes of other elements such as ^15^N, ^34^S, or ^37^Cl in hydroponic cultures can also support sum formula elucidation (Engelsberger et al., 2006; Giavalisco et al., 2011; Dersch et al., 2016).

For the study presented here, comparison of conventional and SIL guided sum formula generation showed, that the number of potential sum formulas roughly decreased by a factor of 5 when the total number of carbon atoms were used instead of relying on the carbon isotopolog ratio of M+1/M (data not shown). Since a lower number of possible sum formulas narrows down the number of possible structures it simplifies metabolite annotation and biological interpretation and selection of the gained hits as has also been described by Giavalisco et al. (2011).

#### Database Search With Masses and Sum Formulas and Verification of Database Hits

As most of the detected metabolites in typical untargeted plant metabolomics experiments cannot be identified, they are subject to putative annotation, which generally includes searching in metabolite repositories. The choice of the used database has substantial influence on the outcome of metabolite annotation. The use of organism-specific databases [for example, PlantCyc (Caspi et al., 2012) or WEIZMASS (Shahaf et al., 2016)] is preferred as the putative annotations are less exhaustive and can be expected to be more reliable than those obtained by the use of more comprehensive, but less biologically oriented databases with up to several millions of compounds (for example ChemSpider (Pence and Williams, 2010); see **Figure 4**). A manual and critical curation of the putative annotations should always be carried out. In this study it is shown that limiting resulting database hits based on knowledge about the incorporation of (substructures of) the tested tracer compounds is an efficient strategy to falsify putative database annotations. However, manual inspection of hundreds to thousands of structure annotations for the detected metabolites is a tedious work and impracticable if the number of compounds gets too large.

Another important issue with respect to metabolite annotation is the presence of multiple isomers. Especially for phenylpropanoids hundreds of isomers exist, which all share the same sum formula and therefore—based on *m/z* and sum formula only—would yield the identical annotations if no reference standard of the compound was available. While classification of those isomers into substance classes may be reasonable, biological interpretation has to be done with great care, especially as isomers may play different biological roles or be affected differently under the chosen experimental conditions. To allow differentiation between isomers, other orthogonal information can be helpful and used together with the presented SIL-strategy. In order to validate or invalidate certain metabolite annotations, evaluation of MS/MS fragment spectra (e.g. comparison with MS/MS databases, *in silico* fragmentation), application of QSRR models [prediction of RTs from chemical properties (Kaliszan, 2007)], or the inspection of mass spectra for the presence/absence of characteristic in-source fragments (e.g. loss of glucose moiety, water, CO_2_, NH_3_ or others) can be considered. Isolation and purification of the compound for subsequent NMR analysis allows definitive structural elucidation of the metabolite but purification of selected compounds is time consuming and hardly applicable on a large scale.

#### Characterization of Unknowns

Even though sum formula generation and database search were successful for most of the metabolites, a considerable number of them still remains unknown and may even miss any kind of database annotation or sum formula prediction using C, H, N, O, S, and P. Based on these elements, it was not possible to calculate putative sum formulas for 198 metabolites (11%) detected in this study. We speculate that these compounds may contain biologically relevant heteroatoms like Ca, Mg, Fe, Co, or Zn which have not been used for sum formula prediction or that other elements are part of the measured adduct ions. Consideration of these elements would enormously increase the number of potential sum formulas thereby making the interpretation even more difficult. Of the 1,729 metabolites detected in the presented experiment, 641 metabolites (37%) could not be annotated with an entry of the ChEBI- or our in-house wheat database.

### Scope and Use of the Presented Workflow Combination

The approach presented here provides unbiased access to the major fraction of unknown metabolites usually present in many biological samples. It offers i) reliable assignment as true wheat-derived metabolites, ii) LC-HRMS data enriched by total number of C-atoms per metabolite ion, iii) assignment to tracer-derived submetabolomes, and iv) also supports the recently developed MS/MS approaches for compound classification by assignment of fragment ions with chemical formulas.

A major purpose of combining GML and TL as described in the presented approach is to generate a global reference inventory of true plant derived metabolites and the comprehensive and unambiguous classification of a subset of these biochemical constituents as members of tracer-derived submetabolomes. All of these metabolites can then be searched for in samples originating from conventional real-world plant metabolomics studies, which do not require any additional labeling effort anymore. The wheat metabolites listed in **Supplementary Table 3** can be used as such a reference template list for other wheat studies.

Ideally, to further enlarge the metabolite coverage between experimental and labeled reference samples, “treatments” of the biological system under labeling conditions can be performed. To include treatment specific metabolites, which may only be formed under the chosen experimental conditions, the type of tracer and, if possible, the treatment parameters should be chosen to reflect those of the biological experiment. If in-house production of the labeled plant samples is not feasible, the isotopically labeled material can be commercially acquired. If the biological organism of interest is not available, a closely related species can be chosen (e.g. a different genotype). While in such a case the overlap of the metabolic constituents will not be complete, still many hundreds to thousands of metabolites will match between the experimental and the isotopically labeled material as demonstrated by Doppler et al. (2016). If there are metabolites that are expected to be relevant but are not part of the automatically generated reference list, those compounds can always be added manually and thus be considered by a targeted search.

Moreover, compared to conventional untargeted approaches, the presented combination of global and tracer based isotopic labeling allows a more comprehensive description of both individual biological samples and single metabolites. Besides the assignment of the number of carbon atoms to every measured metabolite ion, a major innovation is the classification of the metabolites into submetabolomes, which offers valuable information for effective filtering of database hits based on characteristic metabolic substructures. The application of the method will be demonstrated in a related manuscript presenting a study on the Phe-related defense of wheat against the mycotoxin and *Fusarium* virulence factor deoxynivalenol (Doppler et al., 2019).

## Data Availability Statement

All datasets generated for this study are included in the article/**Supplementary Material**.

## Author Contributions

Contributed to the conception and design of the study: MD, BK, CB, RK, RS. Performed the experiments: MD, BK, AK. Helped in setting up the plant labeling: AK, HB, ML, RS. Performed the measurements: MD, BK, JR. Evaluated data: CB, MD. All authors contributed writing the manuscript, read and approved the final version.

## Funding

The authors want to thank the Austrian Science Fund (projects SFB Fusarium F3715 and F3711) and the Provincial Government of Lower Austria (projects NoBiTUM, OMICS 4.0).

## Conflict of Interest

The authors declare that the research was conducted in the absence of any commercial or financial relationships that could be construed as a potential conflict of interest.

## Supplementary Material

Supplementary Datasheet 1 Collection of acquired MS/MS fragmentation spectra.
