# Supplementary figures and images for: Stable Isotope–Assisted Plant Metabolomics: Combination of Global and Tracer-Based Labeling for Enhanced Untargeted Profiling and Compound Annotation
Source: Front Plant Sci. 2019 Oct 25;10:1366. doi: 10.3389/fpls.2019.01366 (PMC6824187; doi:10.3389/fpls.2019.01366)

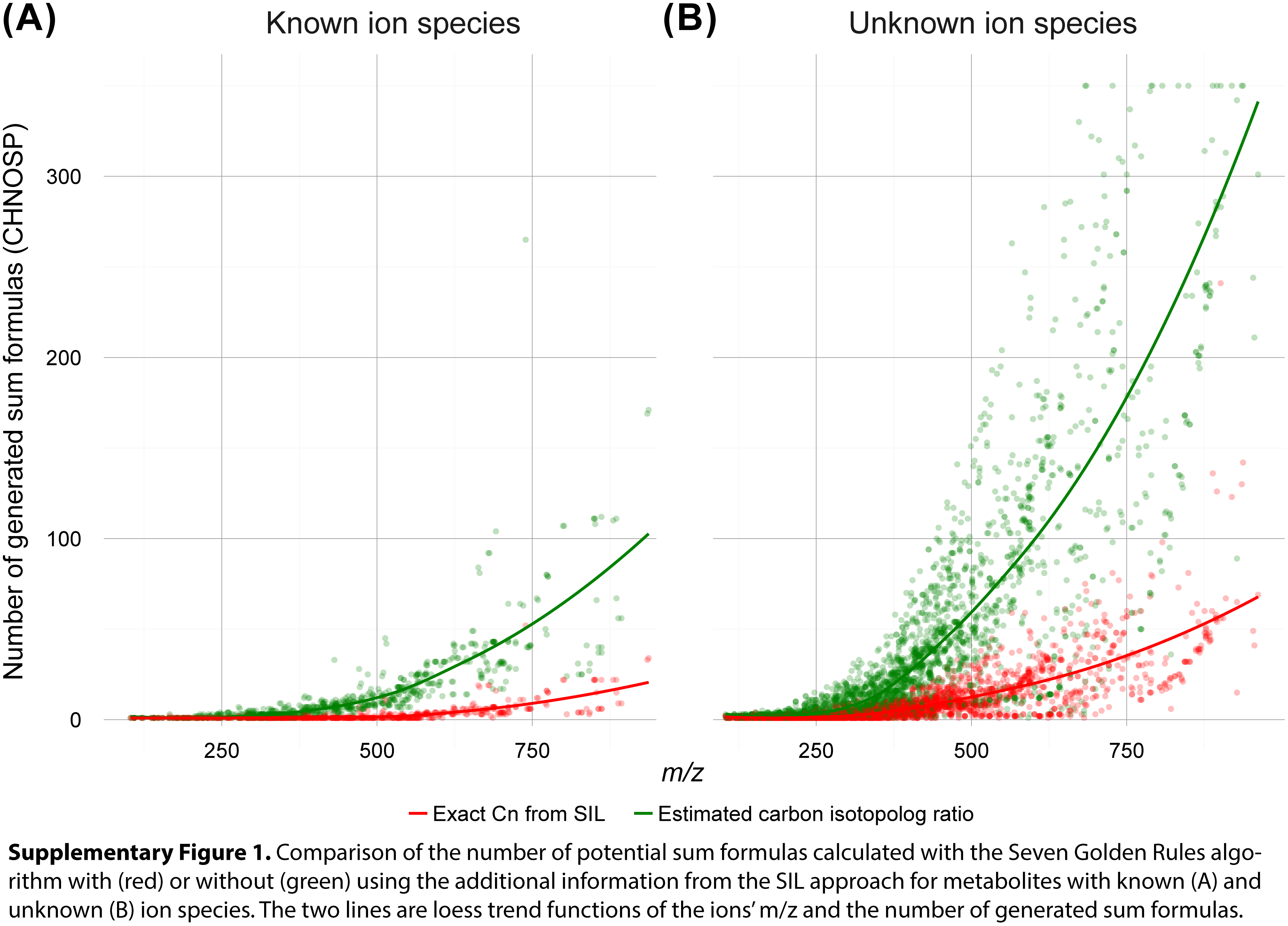

Supplement: Supplementary file 2 [file Image_1.tif]
